# Sensitivity, Specificity and Predictive Values of Molecular and Serological Tests for COVID-19: A Longitudinal Study in Emergency Room

**DOI:** 10.3390/diagnostics10090669

**Published:** 2020-09-03

**Authors:** Zeno Bisoffi, Elena Pomari, Michela Deiana, Chiara Piubelli, Niccolò Ronzoni, Anna Beltrame, Giulia Bertoli, Niccolò Riccardi, Francesca Perandin, Fabio Formenti, Federico Gobbi, Dora Buonfrate, Ronaldo Silva

**Affiliations:** 1 Department of Infectious, Tropical Diseases and Microbiology, IRCCS Sacro Cuore Don Calabria Hospital, Negrar di Valpolicella, 37024 Verona, Italy; zeno.bisoffi@sacrocuore.it (Z.B.); michela.deiana@sacrocuore.it (M.D.); chiara.piubelli@sacrocuore.it (C.P.); niccolo.ronzoni@sacrocuore.it (N.R.); anna.beltrame@sacrocuore.it (A.B.); giulia.bertoli@sacrocuore.it (G.B.); niccolo.riccardi@sacrocuore.it (N.R.); francesca.perandin@sacrocuore.it (F.P.); fabio.formenti@sacrocuore.it (F.F.); federico.gobbi@sacrocuore.it (F.G.); dora.buonfrate@sacrocuore.it (D.B.); ronaldo.silva@sacrocuore.it (R.S.); 2 Department of Diagnostics and Public Health, University of Verona, 37134 Verona, Italy

**Keywords:** SARS-CoV-2, diagnosis, serology, RT-PCR, accuracy

## Abstract

Background: We assessed the sensitivity, specificity and positive and negative predictive value (PPV and NPV) of molecular and serological tests for the diagnosis of SARS-CoV-2 infection. Methods: A total of 346 patients were enrolled in the emergency room. We evaluated three Reverse Transcriptase-real time PCRs (RT-PCRs) including six different gene targets, five serologic rapid diagnostic tests (RDT) and one ELISA. The final classification of infected/non-infected patients was performed using Latent Class Analysis combined with clinical re-assessment of incongruous cases. Results: Out of these, 24.6% of patients were classified as infected. The molecular test RQ-SARS-nCoV-2 showed the highest performance with 91.8% sensitivity, 100% specificity, 100.0% PPV and 97.4% NPV respectively. Considering the single gene targets, *S* and *RdRp* of RQ-SARS-nCoV-2 had the highest sensitivity (94.1%). The in-house *RdRp* presented the lowest sensitivity (62.4%). The specificity ranged from 99.2% for in-house *RdRp* and *N2* to 95.0% for *E*. The PPV ranged from 97.1% of *N2* to 85.4% of *E* and the NPV from 98.1% of *S* to 89.0% of in-house *RdRp*. All serological tests had < 50% sensitivity and low PPV and NPV. VivaDiag IgM (RDT) had 98.5% specificity, with 84.0% PPV, but 24.7% sensitivity. Conclusion: Molecular tests for SARS-CoV-2 infection showed excellent specificity, but significant differences in sensitivity. Serological tests have limited utility in a clinical context.

## 1. Introduction

As of today (27 August 2020), the Severe Acute Respiratory Syndrome Coronavirus 2 (SARS-CoV-2) has infected 23.980.044 individuals, caused 820.763 deaths, and has spread to virtually all countries [1]. Italy has been the first affected country in Europe and one of the most affected worldwide. Although an unprecedented amount of basic and clinical research has been devoted, so far, to this infection, and a few clear lessons have been learned [2], many unsolved issues remain on pathogenetic, immunological and clinical aspects [3,4].

The optimal diagnostic strategy for clinically suspected (symptomatic) cases is not entirely defined, either.

The diagnosis of SARS-CoV-2 infection is based on standardized molecular methods, usually performed on nasal/pharyngeal swabs [5]. However, the accuracy of the different methods has yet to be properly assessed. Sensitivity, for instance, depends on the method itself [6], the correct execution of the nasal and pharyngeal swab [7], and also the passage of time since exposure and onset of symptoms [8,9,10]. False-negative results may cause mismanagement and nosocomial or community transmission [11,12]. False-positive results imply the risk for a patient suffering from another condition to be erroneously admitted to a Coronavirus Disease (COVID) unit, or quarantined at home, besides triggering a complex but useless contact tracing [13].

Many antibody-based tests, including rapid diagnostic tests (RDTs), have been developed [14,15,16,17], marketed and some have already been evaluated in retrospective studies [18,19]. Most RDTs can be performed by a simple finger prick and the result is available in a brief time-lapse. However, the delay between onset of symptoms and detectability of antibodies obviously hampers the sensitivity of RDTs in case of recent infections, thus their diagnostic value at symptoms onset is disputable [20,21,22,23,24,25]. The main use of all serologic tests is now restricted to screening and epidemiologic purposes. It has been suggested, however, that an RDT in combination with a Reverse Transcriptase-real time PCR (RT-PCR) might be useful in clinical practice, too; however, no supporting data have been yet provided on this hypothesis [6].

### Study Objectives

The primary objective of this study was to assess sensitivity, specificity and predictive values of three widely used RT-PCRs, with six different gene targets, for the diagnosis of COVID-19 in clinically suspect cases. The secondary objective was to define whether any of the six serologic tests, five IgG-IgM rapid diagnostic tests (RDT) and an ELISA IgA-IgG test, may be of diagnostic usefulness.

## 2. Materials and Methods

This paper refers to STARD guidelines [26] for the reporting of diagnostic test accuracy. The assessment was carried out using the statistical technique of Latent Class Analysis. 

### 2.1. Type of Study

Observational, prospective diagnostic study. Data collection was planned before performing the index tests and the reference standard tests. 

The study was performed at IRCCS Sacro Cuore Don Calabria Hospital, Negrar, a reference Institute for Infectious and Tropical Diseases in Italy. Patients were enrolled at the first diagnostic workout at the emergency room (ER). 

### 2.2. Study Cohort and Participant Recruitment

All consecutive patients presenting to the ER with clinical suspicion of COVID-19 and submitted to diagnostic tests for SARS-CoV-2 were eligible. The essential clinical and laboratory data were recorded in an electronic Case Report Form (e-CRF). Enrolment continued until completion of the required sample size.

Inclusion criteria: Adult male and female patients consenting to participation in the study and to the donation of biological samples.

Exclusion criteria: Missing or inadequate samples.

### 2.3. Test Methods 

#### 2.3.1. Index Tests 

All the index tests were performed on samples consecutively collected and stored at −80 °C: nasal/pharyngeal swabs for molecular tests and serum for serologic methods.

(a) Molecular tests (RT-PCR)

1. RealQuality RQ-SARS-nCoV-2 assay (cod. RQ-130, AB Analitica, Padova, Italy), targeting the *spike protein* gene (*S*) and the *RNA-dependent RNA polymerase* gene (*RdRp*).

2. CDC 2019-Novel Coronavirus (2019-nCoV) Real-Time RT-PCR Diagnostic Panel (https://www.fda.gov/media/134922/download), targeting the *nucleocapsid protein* gene (*N*), regions *N1* and *N2*.

3. In-house RT-PCR protocol, targeting the *envelope protein* gene (*E*) in the first-line screening assay, followed by confirmatory testing with the *RdRp* gene (same gene as in RQ-130, AB Analitica, but with different target regions) [27]. 

(b) IgG/IgM immune chromatographic RDT

4. 2019–nCoV IgG/IgM Rapid Test Cassette (JusCheck, Acro Biotech, Rancho cucamonga, CA, USA)

5. COVID-19 IgG/IgM Rapid Test Cassette (Femometer Hangzhou Clongene Biotech, Hangzhou, China) 

6. COVID-19 IgG/IgM Rapid Test (Prima Professional, Balerna, Switzerland)

7. VivaDiag 2019-nCoV IgG/IgM rapidTest (VivaCheck Biotech, Hangzhou, China)

(c) IgG/IgM immunofluorescence RDT

8. DiaGreat 2019-nCoV IgG/IgM antibody Determination Kit (Nuclear Laser Medicine, Milano, Italy). 

(d) IgG/IgA ELISA test

9. Anti-SARS-CoV-2 ELISA IgA/IgG (Euroimmun, Lübeck, Germany). 

The test methods are described in detail in the Supplementary Materials.

#### 2.3.2. Blinding 

Each test was executed by experienced lab personnel independently. The lab professionals were not aware of the clinical data of the subjects and did not know in advance the results of any other test.

#### 2.3.3. Reference Standard based on Latent Class Analysis (LCA)

LCA is the preferred method for evaluating a diagnostic test in the absence of a gold standard [28,29,30,31,32] Typically for SARS-CoV-2 infection, no single test can be considered as a gold standard. Tests based on RT-PCR are highly specific, but their sensitivity may not be optimal. The LCA method is summarized in the section “Statistical Methods and Analysis”.

#### 2.3.4. Evaluation Criteria of Molecular Tests Accuracy

Each of the three molecular tests is based on two gene targets, both of which are required to be positive in order to diagnose the infection [27] (https://www.fda.gov/media/134922/download). At first, a restrictive analysis was performed based on this criterion, in which indeterminate results (only one positive gene target) were classified as negative. However, further analysis of accuracy was also performed on single gene targets.

### 2.4. Statistical Methods and Analysis

The sample size calculation was based on the desired 10% width of the 95% exact confidence intervals around the point estimates and on a minimal acceptable sensitivity of 95% and identical specificity. To assure an adequate sample size, 94 patients with SARS-CoV-2 infection and as many negatives were needed. Based on the trend observed in the weeks before study onset, since we expected a proportion of infections of about 25%, we estimated an enrollment of 376 subjects. We planned to enroll 400 subjects to account for possible altered or invalid samples.

Demographic and clinical data were summarized using descriptive statistics and measures of variability and precision. All parameters were reported with 95% confidence intervals (CI). For proportions, the exact Clopper–Pearson CI was computed. 

Diagnostic test results were presented in contingency tables where patients’ disease status was inferred based on probabilistic models using LCA. This is a statistical method used to classify unobserved groups in a population based on a chosen set of indicators. In LCA it is assumed that the true patient condition is unknown (i.e., is a latent class) [32], which can be related to a set of diagnostic test results, clinical and paraclinical variables, through latent class models (LCM). Each class corresponds to a possible condition of the patient, thus a two-class model will classify patients as presumably having/not having the condition, while a three-class model will identify the third group of patients of uncertain classification. The best model and number of classes are chosen based on appropriate statistic methods such as Akaike’s information criterion (AIC) or likelihood ratio test.

Computerized medical records of patients with an uncertain diagnosis according to LCA were reviewed (including all tests repeated in the following days, if any) in order to obtain a reasonably certain diagnosis. Sensitivities, specificities and predictive values of the index tests were calculated based on this final diagnosis.

Data analyses were performed using SAS software, version 9.4 (SAS Institute, Inc., Cary, NC, USA). The statistical significance level was fixed at 0.05. LCMs were built using the LCA procedure with parameters estimated by maximum likelihood using the Expectation-maximization algorithm. A rho prior of strength 1 was used when needed, to avoid estimations on the boundary of the parameter space. Missing values on any diagnostic tests were handled by the LCM. Standard procedures were used for the verification of the assumption of conditional independence between diagnostics tests. To reproduce results, use seed = 1979 in proc LCA.

Further details on the method and the tested models are available in the Supplementary Materials.

The Composite Reference Standard (CRS) is an alternative method for assessing test accuracy when a gold standard is missing. Exploratory analyses were also carried out using CRS for the classification of the study subjects. They are also reported in Supplementary Materials. 

## 3. Results

**Figure 1:**
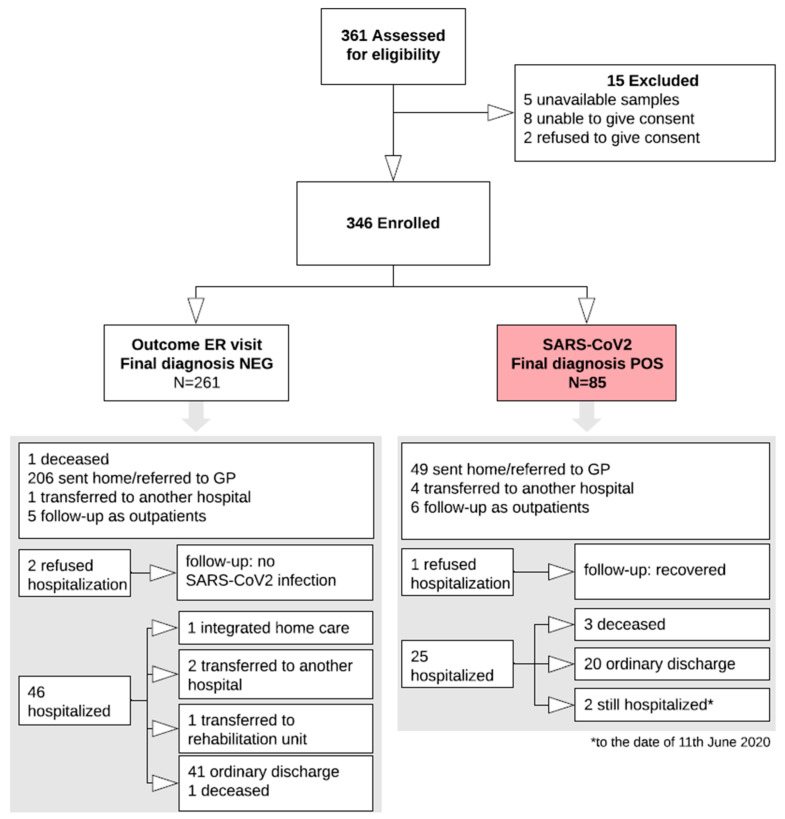
Study Flow chart. The white arrows indicate the study flow, up to the final classification of 85 subjects with the infection (red box) and 261 without the infection. The two grey arrows point to the two boxes resuming all the available details and outcomes of the two groups. ER, emergency room; NEG, negative; POS, positive; GP, general practitioner.

**Figure 2:**
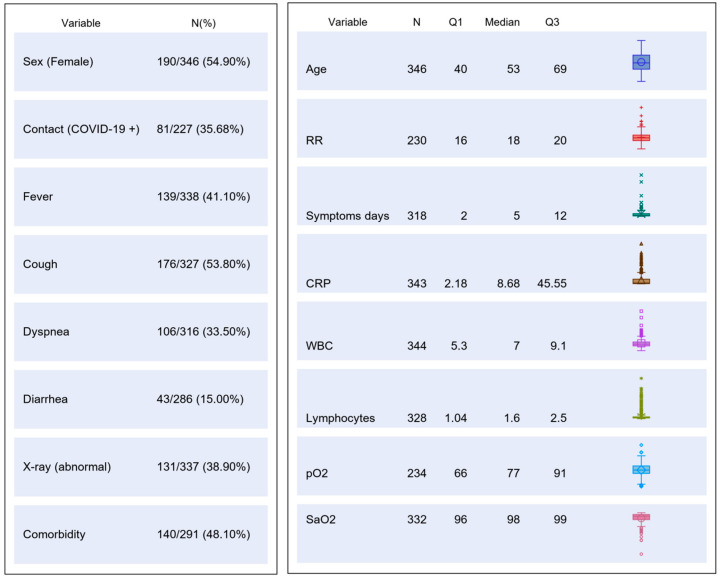
Table chart on main demographic, clinical, laboratory and imaging characteristics of the study population. CRP, C reactive protein; RR, respiratory rate; WBC, white blood cells; pO2, partial pressure of oxygen; SaO2, oxygen saturation.

The study was carried out from 1 March to 9 May 2020. A total of 346 patients were consecutively enrolled (Study Flow Chart, Figure 1). Their main demographic, clinical, laboratory and imaging characteristics are summarized in Figure 2.

Clinical management was based on the result of the molecular test used at ER. 

### 3.1. Latent Class Analysis (LCA)

**Figure 3:**
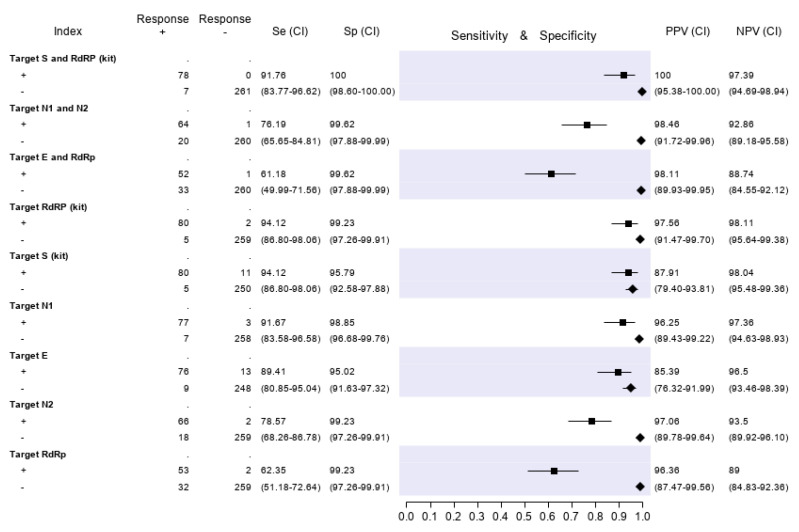
Sensitivity (filled squares), specificity (filled rhombus) and predictive values of three molecular tests and of single gene targets, according to the final classification (Infected = 85, not infective = 261). “Target S and RdRp (kit)” corresponds to the RealQuality RQ-SARS-nCoV-2 assay (cod. RQ-130, AB Analitica, Italy), targeting the *spike protein* gene (*S*) and the *RNA-dependent RNA polymerase* gene (*RdRp*). “Target N1 and N2” corresponds to the CDC 2019-Novel Coronavirus (2019-nCoV) Real-Time RT-PCR Diagnostic Panel, targeting the *nucleocapsid protein* gene (*N*), regions *N1* and *N2*. “Target E and RdRp” corresponds to the *in-house* RT-PCR protocol, targeting the *envelope protein* gene (*E*) and the *RdRp*. +, positive result; −, negative result; Se, sensitivity; Sp, specificity; PPV, positive predictive value; NPV, negative predictive value.

Based on the best fitted LCA model (with three LCA classes), 332 of 346 patients (96%) could be classified as infected or non-infected with virtual certainty. The remaining 14 subjects (4%) could not be definitely attributed to either the infected or uninfected group. The computerized medical records of the latter patients were reviewed (including all tests repeated in the following days, if any) in order to reach a reasonably certain diagnosis. Three patients, for whom the final diagnosis remained doubtful after reassessment, were tested with an additional ELISA Anti-SARS-CoV-2 IgG test (Abbott), performed six to eight weeks after the first diagnosis. The three of them resulted IgG negative and were then were finally classified as non-infected. Furthermore, an assessment of all medical records was performed for patients with at least one discordant gene target result. Finally, 85 out of 346 patients (24.6%) were classified as infected and 261 (75.4%) as non-infected. Based on these denominators and applying the restrictive criterion (both gene targets required to be positive to define a case of infection), the test accuracy results are summarized in Figure 3. The molecular test with the highest sensitivity was RQ-SARS-nCoV-2 (91.8%, C.I. 83.8–96.6), followed by CDC 2019-nCoV (76.2%, C.I. 65.7–84.8) and by in-house primary reference test targeting *E-RdRp* (61.2%, C.I. 50.0–71.6). The specificity was 100% for RQ-SARS-nCoV-2 (C.I. 98.6–100.0) and 99.6% for the other two tests (C.I. 97.9–100.0). The test with the highest PPV and NPV was, again, RQ-SARS-nCoV-2 (100.0%, C.I. 95.4–100.0 and 97.4%, C.I. 94.7–98.9, respectively), followed by CDC 2019-nCoV (98.5%, C.I. 91.7–100.0 and 92.9%, C.I. 89.2–95.6) and by *E-RdRp* test (98.1%, C.I. 89.9–100.0 and 88.7%, C.I. 84.6–92.1).

The results of analyses on single gene targets are also reported in Figure 3. The assays with the highest sensitivity were those targeting *S* and *RdRp* of the RQ-SARS-nCoV-2 (both with sensitivity 94.1%, C.I. 86.8-98.1). The in-house *RdRp* was the target with the lowest sensitivity (62.4%, C.I. 51.2–72.6). The specificity ranged from 99.2% (C.I. 97.3–99.9) for in-house *RdRp* and *N2* to 95.0% (C.I. 91.6–97.3) for gene *E*. The PPV ranged from 97.1% (C.I. 89.8–99.6) of *N2* gene to 85.4% (C.I. 76.3–92.00) of *E* gene and the NPV from 98.1% (C.I. 95.5–99.4) of gene *S* to 89.0% (C.I. 84.8–92.4) of in-house *RdRp*. 

### 3.2. Concordance among the Six Gene Targets

**Figure 4:**
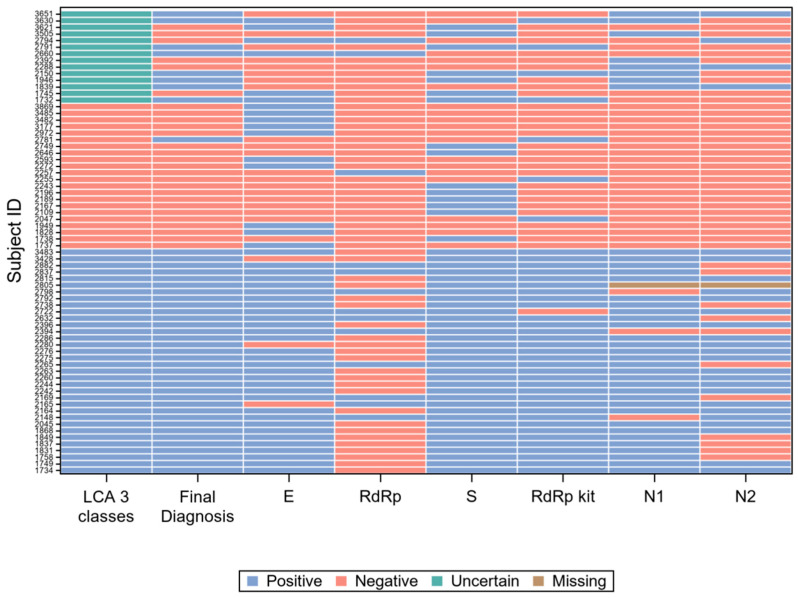
Visual representation of the 70 records with at least one discordant gene target result.

For 42 of the 85 subjects with a positive final diagnosis (49%), all gene target results were concordant positive, while for 234 of the 261 subjects with a negative final diagnosis (90%), they were concordant negative. The 70 records with at least one discordant result are graphically represented in Figure 4.

### 3.3. Evaluation of the Serologic Tests

**Figure 5:**
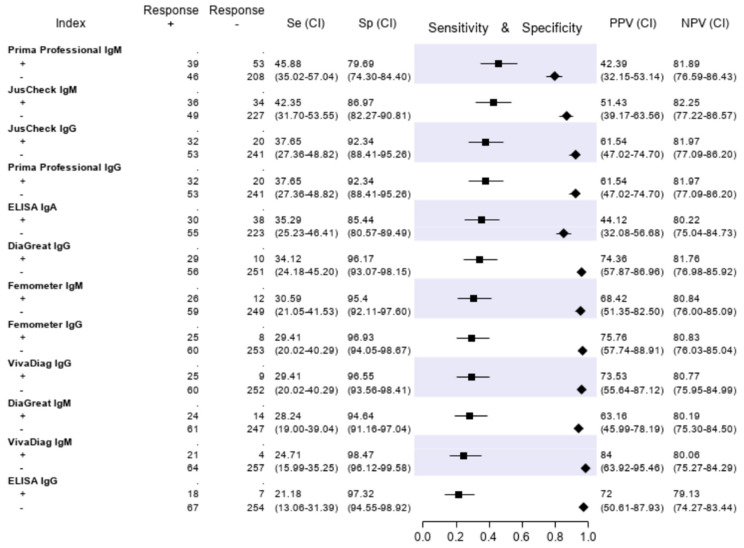
Sensitivity (filled squares), specificity (filled rhombus) and predictive values of six antibody tests according to the final classification (Infected = 85, not infective = 261).

**Figure 6:**
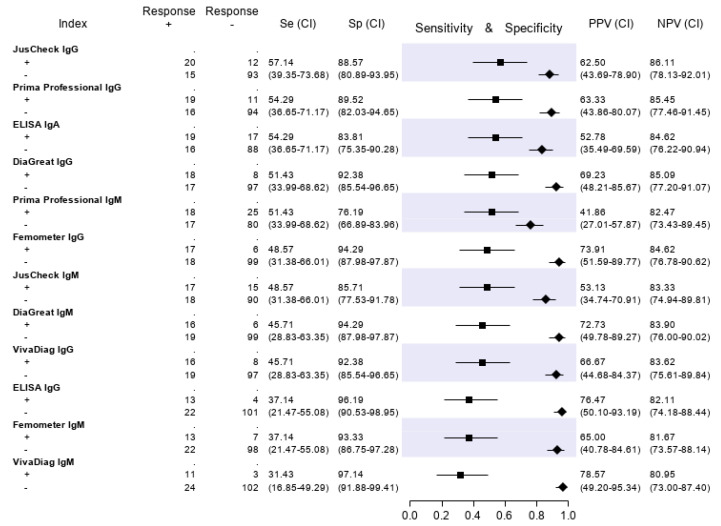
Test accuracies using a subset of 140 patients with a symptom duration of ≥ 7 days. Sensitivity (filled squares), specificity (filled rhombus) and predictive values of six antibody tests according to the final classification.

The results of the serologic tests are reported in Figure 5. Briefly, the sensitivity ranged from 45.9% (Prima Professional IgM) to 21.2% (ELISA IgG); the specificity, from 98.5% (VivaDiag IgM) to 79.7% (Prima Professional IgM); the PPV, from 84.0% (VivaDiag IgM) to 44.1% (ELISA IgA); the NPV, from 82.3 (JusCheck IgM) to 79.1% (ELISA IgG). An exploratory analysis was made on a subgroup of patients with a symptom duration of ≥7 days. For this exploratory analysis, we considered a subgroup of 140 of 346 (40.46%) patients who reported respiratory symptoms, difficulty in breathing, fever, diarrhea, dyspnea or cough for at least seven days. Test accuracies are presented in Figure 6. The sensitivity of serological tests improved, ranging from 57.1% of JusCheck IgG, to 31.4% of VivaDiag IgM, but remained low for all tests. The specificity slightly reduced, ranging from 97.1% (VivaDiag IgM) to 76.2% (Prima Professional IgM). 

## 4. Discussion

This is, to our knowledge, the first formal longitudinal accuracy study of both molecular and serologic tests for the diagnosis of SARS-CoV-2 infection in suspected COVID-19 patients. The combined molecular tests (targeting two genes) showed significant differences in sensitivity, which was >90% only for RQ-SARS-nCoV-2. This raises concern on the current protocols for COVID-19 diagnosis, as most require at least two different SARS-CoV-2 genomic regions to be concordantly positive in order to classify a subject as infected [6,7,27,33,34,35]. Actually, early in the course of the epidemic, we realized that a number of patients who resulted positive at the first-line screening test (*E* target) but negative at the confirmatory test (*RdRp* target), and who would have been then classified as non-infected with SARS-CoV2, were most probably true positives, and we started managing patients accordingly. Interestingly, in accordance with our practice, current US FDA recommendations consider a single positive gene target as sufficient to validate the performance of RT-PCR assays for the diagnosis of COVID-19 (https://www.fda.gov/medical-devices/emergency-situations-medical-devices/faqs-testing-sars-cov-2).

However, the concordance among molecular gene targets was far from perfect. Figure 4 resumes the results of all single gene targets, compared with the patient classification according to LCA and with the final diagnosis, on the 70 samples (20% of the total) with at least one discordant result. It is immediately apparent from Figure 4 that the gene target *RdRp* of the in-house protocol [27] (“*RdRp*” in the figure) accounts for a large proportion of the results discordant with the final diagnosis, while the same gene (with different molecular targets) of RealQuality RQ-SARS-nCoV-2 assay (“*RdRp* kit” in the figure) was the single gene that minimized discordant results.

Clearly, however, whatever the test used, a variable proportion of truly infected patients may be missed, and patients with a high clinical suspicion should be carefully considered, even after a negative test result. Looking back at our study population, we realized that three out of five patients who had been initially wrongly diagnosed as negative, were in fact rightly managed as COVID-19 patients, due to a clinical suspicion and consequent repetition of the test in subsequent nasal/pharyngeal swabs. However, two COVID-19 patients were incorrectly diagnosed and managed, as the infection was only demonstrated in retrospect. 

The specificity was very high, expectedly, for all molecular tests when using the restrictive diagnostic criterion. When using the “relaxed” criterion of relying on a single gene target, the increased sensitivity is unsurprisingly mirrored by some loss in specificity. When dealing with clinically suspect cases in a phase of intense transmission, high sensitivity is required, as missing a case would have serious consequences [13]. Also, in the presence of high clinical suspicion or pre-test probability, the positive predictive value of a test is obviously higher than in a screening context. Thus, recommendations on the correct interpretation of test results should be tailored to the clinical and epidemiological context. When the tests are used on suspect cases of COVID-19, in a phase of intense transmission, the application of the relaxed criterion is amply justified. However, when the same tests are used for screening purposes in a phase of low/very low viral circulation, relying on a “single-gene” approach would result in a higher proportion of false-positive results.

None of the serologic tests showed acceptable sensitivity nor specificity, confirming previous reports claiming that serologic tests are unsuitable for clinical use on acutely ill patients and that their deployment should be limited to epidemiologic purposes [20,36,37,38,39]. Our results also show that when stratifying the patients based on the time elapsed since the symptom onset, for those with one week or more of symptom duration the sensitivity of all serologic tests is higher, but still far from satisfactory. Given that molecular tests are not 100% sensitive either, a combination of molecular plus serologic tests could be devised. However, our study does not provide enough data to support this strategy. Obviously, paired serologic tests looking for seroconversion would certainly increase the sensitivity of serologic tests but with little, if any, clinical use.

### 4.1. Strengths

The study was conducted closely adhering to STARD guidelines. Moreover, in order to cope with the lack of a gold standard, the main analyses were carried out using LCA, on the condition that the chosen models properly fitted the data. This study on a comparatively large cohort of patients suggests possible alternatives to current diagnostic protocols, in order to avoid the potentially dangerous premature exclusion of a case of infection.

### 4.2. Limitations 

The sample size was slightly lower than the calculated number of 376 patients, due to the rapid decrease of new cases in the last period of the investigation. However, samples from almost all patients recruited were subsequently analyzed as there were no altered or invalid specimens, reaching a final number of 346 patients, which was close to the planned sample size. 

Despite the longitudinal study design, some clinical data were missing for a number of patients, which also reflects the inherent difficulties in performing clinical studies in emergency situations. However, for most variables included in the model, the data set was sufficiently complete.

## 5. Conclusions

The molecular tests here evaluated demonstrated significant differences in sensitivity. For molecular diagnostic purposes, accepting positive results in any single gene target appears justified for cases with clinical suspicion of COVID-19 in an ER. Conversely, a confirmation of the diagnosis, based on the positivity of multiple genomic regions, might be more appropriate when the test is deployed for screening purposes in a phase of low/very low viral circulation. 

The serologic tests included in this study did not demonstrate suitable sensitivity for clinical use on acutely ill patients.

## Data Availability

The data supporting the findings of this study are included in this published article (and its Supplementary Material).

## Supplementary Materials

The Supplementary Materials are available online at https://www.mdpi.com/2075-4418/10/9/669/s1.
