# Supplementary material for: Sensitivity, Specificity and Predictive Values of Molecular and Serological Tests for COVID-19: A Longitudinal Study in Emergency Room
Source: Diagnostics (Basel). 2020 Sep 3;10(9):669. doi: 10.3390/diagnostics10090669 (PMC7555224; doi:10.3390/diagnostics10090669)
Supplement: Supplementary file 1 [file diagnostics-10-00669-s001.pdf]

diagnostics

Supplementary materials

Sensitivity, specificity and predictive values of molecular and serological tests for COVID-19: A longitudinal study in emergency room

Authors: Zeno Bisoffi, Elena Pomari, Michela Deiana, Chiara Piubelli, Niccolò Ronzoni, Anna Beltrame, Giulia Bertoli, Niccolò Riccardi, Francesca Perandin, Fabio Formenti, Federico Gobbi, Dora Buonfrate and Ronaldo Silva.

Table of contents

Supplement to: ... 1

Sensitivity, specificity and predictive values of molecular and serological tests for COVID-19. ... 1

A longitudinal study in emergency room ... 1

1. List of investigators ... 2
2. Technical details of the tests and reasons for the choice of the Primary Reference Standard (PRS) ... 2
3. Reference Standard tests ... 4

Indeterminate results of serological tests ... 4

4. Latent Class Analysis (LCA) ... 5
   - 4.1. LCA – Model 1 – 6 molecular tests ... 6
   - 4.2. LCA – Model 2 – 6 molecular tests + clinical and laboratory covariates ... 6
   - 4.3. LCM M1 vs M2 using 309 observations ... 6
   - 4.4. LCA 3 classes model ... 6
5. Final diagnosis ... 7
6. Composite Reference Standard (CRS) ... 7
   - 6.1. Conservative CRS ... 7
   - 6.2. NON conservative Composite Reference Standard (nCRS) ... 7

1. List of investigators

IRCCS Sacro Cuore Don Calabria Hospital (Italy)

Zeno Bisoffi, Elena Pomari, Michela Deiana, Chiara Piubelli, Niccolò Ronzoni, Anna Beltrame, Giulia Bertoli, Niccolò Riccardi, Francesca Perandin, Fabio Formenti, Federico Gobbi, Dora Buonfrate, Ronaldo Silva, Stefano Tais, Monica Degani, Marco Prato, Flavio Stefanini, Arjan Qefalia, Flavio Coato, Francesca Tamarozzi, Antonio Mori, Fabio Chesini, Giulia La Marca, Barbara Pajola.

2. Technical details of the tests and reasons for the choice of the Primary Reference Standard (PRS)

Index tests

1) RealQuality RQ-SARS-nCoV-2 assay (cod. RQ-130, AB Analitica, Italy).
2) CDC 2019-Novel Coronavirus (2019-nCoV) Real-Time RT-PCR Diagnostic Panel.
3) In-house RT-PCR protocol performed on nasal/pharyngeal swabs, targeting the *envelope protein* gene (*E*) in the first-line screening assay, followed by confirmatory testing with the *RdRp*
4) 2019–nCoV IgG/IgM Rapid Test Cassette (JusCheck, Acro Biotech, USA)
5) COVID-19 IgG/IgM Rapid Test Cassette (Femometer Hangzhou Clongene Biotech, China)
6) COVID-19 IgG/IgM Rapid Test (Prima Professional, Switzerland)
7) VivaDiag 2019-nCoV IgG/IgM rapid Test (VivaCheck Biotech, China)
8) DiaGreat 2019-nCoV IgG/IgM antibody Determination Kit (Nuclear Laser Medicine, Italy).
9) Anti-SARS-CoV-2 ELISA IgA/IgG (Euroimmun, Italy).

Molecular tests

For molecular tests, the remaining RNA aliquots after performing the reference standard test were stored at -80°C until used for the following additional tests of reverse transcriptase real time PCR (RT-PCR):

-[**1**]: the RT-PCR reaction mix was prepared combining 17.5μL of the Real time Mix, 2.5μL of RT Enzyme mix and 10μL of RNA from the tested subject (or positive control), for a total volume of 30μL per reaction. The thermal protocol was 10min at 48°C, 10min at 95°C, followed by 45 cycles at 95°C for 15sec and 60°C for 1min.

-[**2**] (https://www.fda.gov/media/134922/download): the RT-PCR reaction mix was prepared combining 6.25μL of TaqMan® Fast Virus 1-Step Master Mix (Thermofisher), 1.5μL of combined Primer/Probe Mix and 10μL of RNA from the tested subject (or positive control), for a total volume of 20μL per reaction. The thermal protocol was 5min at 50°C, 20sec at 95°C, followed by 45 cycles at 95°C for 3sec and 55°C for 30sec. As internal control (IC), human βActin target was used, according to the protocol developed in house for the diagnostic test. Briefly, the real-time PCR mix was performed adding 5μL of TaqMan® Fast Virus 1-Step Master Mix (Thermofisher), 1.5μL of combined Primer/Probe Mix, 0,5μL of Bovine Serum Albumin (BSA) and 5μL of RNA for a final volume of 25μL.

-[**3**] in-house RT-PCR protocol performed on nasal/pharyngeal swabs, targeting the *envelope protein gene* (*E*) in the first-line screening assay, followed by confirmatory testing with the *RdRp gene*[1]. As internal control (IC), the human β-Actin target was used. The procedure was cross-validated with the Regional Reference laboratory (Department of Microbiology, University Hospital of Padua). Briefly, nasal/oropharyngeal swabs were collected from subjects at admission. RNA was isolated in accordance with our routine laboratory practice by the MagnaPure LC.2 instrument (Roche Diagnostic), using the MagNA Pure LC RNA Isolation Kit - High Performance (Roche), according to the manufacturer's instructions. Eluted RNA was analysed by the routine *in-house* RT-PCR protocol for the SARS-CoV-2 as previously reported[1]. The remaining RNA aliquots were stored at -80°C until used for the following additional molecular tests (as described above). The sample was considered positive for SARS-CoV-2 only if the signal of all the targets was present. If the IC signal was present and the signal of the other targets were undetectable, the sample was considered negative. In all the other cases, the sample was considered indeterminate.

Serological tests

Blood was collected from subjects at admission. Serum was separated from blood as soon as possible, aliquoted and stored at -80°C until used. All the serological tests were performed on serum samples, according to the manufacturers' instructions, as summarized below.

- Immunochromatographic Tests [**4-7**]: 10μL of serum was transferred to the specimen well, then 2 drops of buffer was added. With the appearance of the colored line(s), the results were read in 10-15min. Results were considered acceptable only when the line in the control region (C) was clearly visible and outcomes were classified as follows: Negative (=0), no colored line in either the IgG (G) or IgM (M) regions; IgG or IgM Weak Positive (=1): one faint colored line in either G or M. IgG and IgM Weak Positive: faint color lines in both G and M; IgG or IgM Positive (=2): clearly visible colored lines in either G or M regions. IgG and IgM Positive (=2 for both IgM and IgG): clearly visible colored lines in both G and M regions. According to the manufacturers, all positive lines (independently on how clearly visible) should be read as positive. To the study purpose, weak positives were classified as indeterminate. For the main analyses, indeterminate results were classified as positive, following the manufacturers' indications. The tests were independently read by two experienced technicians and classified according to the score assigned by the readers (0, 1 or 2). Discordant results were read by a third reader and finally classified accordingly.

- Fluorescence immunochromatography assay [**8**]: 10μL of serum was added to the provided reaction buffer and shaken for 15 sec., then 80μL of mixed sample were transferred to the specimen well of a strip. After 15 min. the strip was loaded into a slot of a dedicated reader and results were printed directly from the The sample was considered negative when the value (U/L) was <0.8, while positive when ≥1. Values between 0.8 and 1 were classified as indeterminate. For each sample one strip for IgM and one for IgG were analyzed.

- ELISA assay [**9**]: microtiter plates with adsorbed, recombinant structural proteins of SARS-CoV-2 were used to detect IgA or IgG. 10μL of serum samples were diluted in 1ml of the provided reaction buffer, then 100μL of mixed sample were transferred to the well plate. After an incubation of 60 min. at 37°C, plates were read on microplate reader ELx8000 (BioTek) at 450nm. The sample was considered negative when OD Ratio (sample's absorbance/calibrator's absorbance) was <0.8, while positive when OD Ratio was ≥1. If the value was between 0.8 and 1 the result was considered indeterminate.

Indeterminate results of the ELISA and of Fluorescence immunochromatography assay were classified as negative.

All PCRs were run on a ABI 7500 FastDx (Thermofisher) or CFX96 (BioRad). The results were interpreted according to the manufacturer's recommendation. Concisely, the sample was considered positive for SARS-CoV-2 only if the signal of all the targets (including IC) was present. If the IC signal was present and the other targets were undetectable, the sample was considered negative. In all the other cases, the sample was considered indeterminate.

Blinding. Each test was executed by experienced lab personnel of the reference laboratory independently. The lab professionals were not aware of the clinical data of the subjects and did not know in advance the results of any other test.

3. Reference Standard tests

Primary Reference Standard (PRS)

The internal, Primary Reference Standard for the evaluation of serologic tests was our in-house RT-PCR protocol performed on nasal/pharyngeal swabs, targeting the *envelope protein gene* (*E*) and the *RdRp gene*[1].

However, the main analyses described in the main paper do not refer to the PRS, for the reasons outlined below.

Rationale for choosing the reference standard methods

In the case of SARS-CoV2, the RT-PCR test, revealing the specific sequences of the virus, may be considered the acceptable gold standard for calculating the sensitivity of index tests, being a test with virtual 100% specificity. The molecular test routinely used at our lab was then used as a reference for the first analysis of accuracy of molecular tests. However, the sensitivity of the RT-PCR cannot be considered to be 100%, for different reasons: in some cases the target sequences could not be revealed because of a too low viral load or an imperfect execution of the swabs. In the case only this "gold standard" is used, classification of discordant results (negative gold standard, positive index test) would be subject to error. Using a composite reference standard (CRS) is one of the alternative methods when a "perfect" gold standard is not available[2,3]. However, this method has some limitations too, and when a CRS is used, its accuracy cannot be defined *"a priori"*[4]. Alternative methods to cope with the lack of a gold standard are latent class models[5]. In the present study, latent class analysis (LCA) was planned as the main analysis, using all the available molecular tests for SARS-CoV2 as well as other, selected, clinical and paraclinical variables.

The accuracy results of the six serologic tests using the PRS are summarized in Figure 1.

Indeterminate results of serological tests

For both ELISA and DiaGreat tests, Values <0.8 were negative, ≥1 were positive. Values between 0.8 and 1 were classified as indeterminate. Those were treated as negative for the study purpose. For immunochromatographic RDTs, according to the manufacturers, only positive or negative results were considered, although positives were graded by the readers with score 1 (weak positive) or 2 (positive) as explained in this Supplement, Chapter 2. Tests with discordant results between the two independent readers were read by a third independent reader. They were finally classified according to the latter reading. Details of discordant results for RDTs are summarized below. Briefly, discordant results among Readers 1 and 2 were resolved by a third Reader. Data are ordered by kappa coefficient

Test	Positive discordant results*	Negative discordant results*	Cohen's kappa coefficient (95% CI)
VivaDiag IgM	0	0	1.0000 (1.0000,1.0000)
JusCheck IgG	0	1	0.9886 (0.9663,1.0000)
VivaDiag IgG	1	0	0.9835 (0.9512,1.0000)
Femometer IgG	1	0	0.9830 (0.9498,1.0000)
Primacheck IgG	0	3	0.9652 (0.9261,1.0000)
JusCheck IgM	4	6	0.9064 (0.8493,0.9635)
Femometer IgM	3	4	0.8873 (0.8050,0.9696)
Primacheck IgM	8	13	0.8343 (0.7659,0.9027)

*according to Reader 1.

Most of discordant results were between 0 (negative) and 1 (weak positive or indeterminate), but a few were also discordant between 0 and 2 (frank positive).

4. Latent Class Analysis (LCA)

LCA was used as the main reference method for the reasons explained above as well as in the main manuscript.

Latent class models (LCM) were built using LCA procedure available in SAS software version 9.4 (SAS Institute, Inc., Cary, NC, USA). LCM parameters were estimated by maximum likelihood using the Expectation-maximization algorithm. A rho prior of strength 1 was used when needed, to avoid estimations on the boundary of the parameters space. Missing values on any diagnostic tests

were handled by the LCM. Models 1 and 2 are two-classes LCM corresponding to whether or not the patient has the disease based on the highest probability assigned to each class. Model 3 is a three-class LCM model allowing for diagnostic uncertainty as and outcome. Models are compared using statistics such as Akaike's information criterion (AIC) or likelihood ratio test.

LCA assumptions and limitations: Our LCA is based on the assumption that the diagnostic tests under evaluation are independent since they have different gene targets or regions. In the absence of a gold standard LCMs provide means of making inference on the patient's disease status by creating, using Pepe and Alonzo 2001 own words, a "consensus golden standard" from several tests[6]; therefore, the number of tests added to the model and individual performances are critical for the model building process. In classical LCA, if a test is 100% sensitive or specific, it cannot alone rule in or out (according to the result) the condition.

4.1. LCA – Model 1 – 6 molecular tests

LCM was set with two latent classes and six molecular tests targeting *genes E*, *RdRp* (2 tests), *N1*, *N2* and *S*. To reproduce results seed=1979 in proc LCA was used. Model diagnostics are presented below.

DEGREES OF FREEDOM	LOG LIKELIHOOD	AIC	BIC	G SQUARED
50	-508.9909629	98.035716263	98.035716263	72.035716263

4.2. LCA – Model 2 – 6 molecular tests + clinical and laboratory covariates

The initial LCMs were set with two latent classes, six molecular tests targeting genes *E*, *RdRp* (2 tests), *N1*, *N2* and *S* and 7 covariates, namely Chest X-Ray (1=Abnormal, 2=Normal), Lymphocytes (1 if <= 0.9 (10^9/L), 2 otherwise), Respiratory Rate (1 if > 20 per min, 2 otherwise), PCR (1 if >= 6 (mg/L), 2 otherwise), pO2 (1 if <= 75 (mmHg), 2 otherwise), SaO2 (1 if <= 94%, 2 otherwise), White blood cells (1 if <= 4 (10^9/L), 2 otherwise), Fever (1 if > 37.5C, 2 otherwise), Comorbidity (1=Yes, No=2), Diarrhea (1=Yes, No=2), Dyspnea (1=Yes, No=2), Contact with person infected of SARS-Cov2 (1=Yes, No=2), Cough (1=Yes, No=2) and Duration of Symptoms (1 if >= 7 days, 2 otherwise). Note that some models had different number of observations because missing values on the covariates making it difficult to compare the full or final model with individual models with just one covariate.

Considering one covariate at a time in the model with six molecular tests, the following resulted statistically significative at 0.1 significance level: Comorbidity (p-value= 0.071953), Respiratory Rate (p-value= 0.00831, odds ratio=2.56709, 95% CI=1.18676 to 5.55290), White blood cells (p-value < 0.0001, odds ratio=16.8827, 95% CI=7.62308 to 37.3899), Lymphocytes (p-value= 0.000 p-value= < 0.0001, odds ratio=3.65742, 95% CI=2.03418 to 6.57598006), PCR (p-value= 0.02175, odds ratio=1.72819, 95% CI=1.01610 to 2.93932), pO2 (p-value= 0.04899, odds ratio=1.64714, 95% CI=0.91204 to 2.97473), Chest X-Ray (p-value= 0.00029, odds ratio=2.47946, 95% CI=1.47889 to 4.15697), SaO2 (p-value= 0.000206, odds ratio=3.06864, 95% CI=1.64708 to 5.71709) and Fever (p-value= 0.03800, odds ratio=1.59683, 95% CI=0.95220 to 2.67787). Covariates from this first assessment were considered in a full model (with 309/346 observations) resulting in 3 significant covariates (at 0.05 significance level): Chest X-Ray (p-value= .006792520), Lymphocytes (p-value= .000170855) and SaO2 (p-value= .006777484). Model's log likelihood was -463.1072791. Proc LCA does no estimate degrees of freedom, AIC, BIC and G squared when covariates are added to the model.

Results are presented in Table 1a. Table 1b presents, for each covariate, the odds of having a positive result giving that Chest X-Ray is abnormal, Lymphocytes <= 0.9 (10^9/L) or SaO2 is <= 94%. In Table 1c LCM M2 results are reported for cases when molecular test are not in full agreement. Discordant results represent 21.68% (67/309).

4.3. LCM M1 vs M2 using 309 observations

To make LCM M1 and M2 comparable, we fitted M1 using the same 309/346 observations used by M2. Model fitting results are showed below.

DEGREES OF FREEDOM	LOG LIKELIHOOD	AIC	BIC	G SQUARED

50	-478.9703947	89.342281071	137.87571767	63.342281071

Using log likelihood ratio test resulted that model M2 is better when compared to M1 (p-value < 0.0001). M2 has also a smaller AIC value.

4.4. LCA 3 classes model

LCM was set with three latent classes and six molecular tests targeting *genes E, RdRp* (2 tests), *N1, N2* and *S*. To reproduce results seed=1979 in proc LCA was used. Model diagnostics are presented below. Results are presented in the manuscript.

DEGREES OF FREEDOM	LOG LIKELIHOOD	AIC	BIC	G SQUARED
43	-492.4169488	78.887688062	155.81646356	38.887688062

Using log likelihood ratio test resulted that model M3 is better when compared to M1 (p-value < 0.0001). M3 has also a smaller AIC value.

5. Final diagnosis

We named "final diagnosis" the following procedure used to classify with the highest certainty the cases as infected or non infected. The medical records of patients belonging to Class 3 (14 patients not attributed to either the infected or non infected groups with certainty or very high probability) were all clinically reviewed in order to be attributed to the infected or non infected group. In order to further check the LCA model, all the records of the patients with at least one discordant target gene result were also clinically reviewed.

The accuracy results based on the final diagnosis are reported in the main manuscript. Of note, among the 56 records belonging to LCA class 1 or 2 that had at least one discordant target gene result, 55 (98%) were confirmed after the clinical revision, and only one that would have been classified as non infected by LCA was finally classified as infected. In Table 2, the patient classification based on the internal PRS is compared with that based on the final diagnosis.

6. Composite Reference Standard (CRS)

As an exploratory analysis, CRS was also used for the patient classification, also in order to compare LCA classification with that of another method that is often used in the absence of a gold standard.

6.1. Conservative CRS

1st step: results from gene targets *E* & *RdRp*, *N1* & *N2* and *S* & *RdRp* were combined using a conservative rule, i.e, for each of the three molecular tests: Positive = both genes positives, Negative = one or both genes negatives.

2nd step: target combinations obtained from 1st step were combined to form the CRS using the following rule: if two pairs of combinations from step 1 were positive, then CRS=Positive; otherwise CRS=Negative.

6.2. NON conservative Composite Reference Standard (nCRS)

1st step: results from gene targets *E* & *RdRp*, *N1* & *N2* and *S* & *RdRp* were combined using a NON conservative rule, i.e, for each of the three molecular tests: Positive = one or both genes positives, Negative = both genes negatives.

2nd step:same as in 2nd step of CRS.

Results are presented in Table 3. In Figure 2 we report the CRS and nCRS results for all the patients with at least one discordant gene target, in comparison with those of the final (LCA-based) classification. Fifteen cases would have been classified differently, if CRS was used as the final reference (all false negative and no false positive). Using nCRS, 9 cases would be discordant with the final classification (4 false positives and 5 false negatives).

Supplementary Tables and Figures

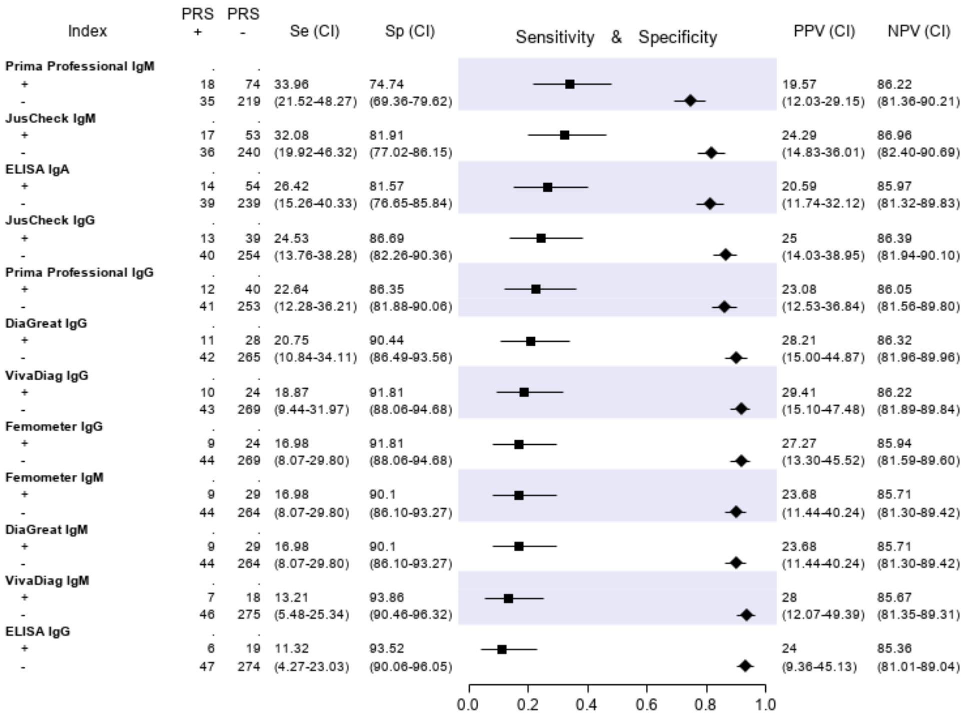

Figure S1. Sensitivity (filled squares), specificity (filled rhombus) and predictive values of 6 antibody tests according to the Primary Reference Standard (Gene E + Gene RdRp: positive=53, negative=293).

Table S1a. Results for LCM M2 with covariates for the combination of molecular test: gene targets *E*, *RdRp*, *N1*, *N2*, *S* and *RdRP* (RQ-SARS-nCoV-2).

Class	Frequency	Percent	Cumulative Frequency
-	231	74.76	
+	78	25.24	309

Table S1b. Odds ratio and 95% confidence intervals for covariates that entered in the final model LCM M2.

Variable	Odds Ratio (95% CI)
Chest X-Ray	1.98936 (1.14383-3.45992)
Lymphocytes	3.01452 (1.64049-5.53940)
SaO2	2.23706 (1.15106-4.34767)

Table S1c. Discordant results among molecular tests and LCM M2 results.

Class	Frequency	Percent	Cumulative Frequency
-	31	46.27	
+	36	53.73	67

Table S2. Contingency table for the PRS and final diagnosis.

	Final Diagnosis		
PRS	+	-	Total
+	52	1	53
-	33	260	293

	Final Diagnosis		
**PRS**	+	-	**Total**
**Total**	85	261	346

Table S3. Results for the combination of molecular test *Gene E*, *RdRp*, *N1*, *N2*, *S* and *RdRP* (RQ-SARS-nCoV-2), using the conservative CRS and nCRS rules.

	**Frequency**	**Percent**	**Cumulative Frequency**
**CRS**			
-	276	79.77	
+	70	20.23	346
**nCRS**			
-	262	75.72	
+	84	24.28	346

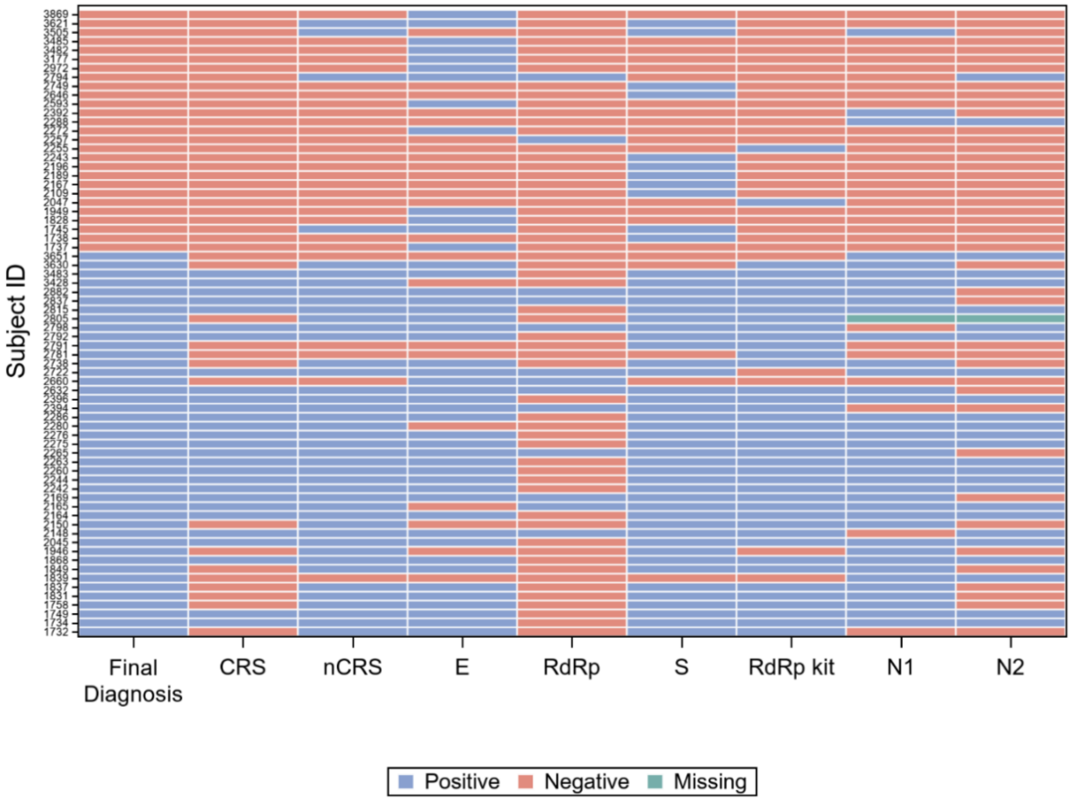

Figure S2. Discordant results among various molecular tests, compared with LCA-based final diagnosis and with CRS and nCRS.

Table 4. STARD-2015-Checklist.

Section & Topic	No	Item	Reported on page #
TITLE OR ABSTRACT			
	**1**	Identification as a study of diagnostic accuracy using at least one measure of accuracy (such as sensitivity, specificity, predictive values, or AUC)	1
ABSTRACT			
	**2**	Structured summary of study design, methods, results, and conclusions (for specific guidance, see STARD for Abstracts)	1
INTRODUCTION			
	**3**	Scientific and clinical background, including the intended use and clinical role of the index test	1;2
	**4**	Study objectives and hypotheses	2
METHODS			
Study design	**5**	Whether data collection was planned before the index test and reference standard were performed (prospective study) or after (retrospective study)	2
Participants	**6**	Eligibility criteria	2

	**7**	On what basis potentially eligible participants were identified (such as symptoms, results from previous tests, inclusion in registry)	2
	**8**	Where and when potentially eligible participants were identified (setting, location and dates)	2; 4
	**9**	Whether participants formed a consecutive, random or convenience series	2
Test methods	**10a**	Index test, in sufficient detail to allow replication	3 and 2; 3 (appendix)
	**10b**	Reference standard, in sufficient detail to allow replication	3 and 4 (appendix)
	**11**	Rationale for choosing the reference standard (if alternatives exist)	4 (appendix)
	**12a**	Definition of and rationale for test positivity cut-offs or result categories of the index test, distinguishing pre-specified from exploratory	3 and 2;3;4 (appendix)
	**12b**	Definition of and rationale for test positivity cut-offs or result categories of the reference standard, distinguishing pre-specified from exploratory	3 and 4 (appendix)
	**13a**	Whether clinical information and reference standard results were available to the performers/readers of the index test	3 and 3 (appendix)
	**13b**	Whether clinical information and index test results were available to the assessors of the reference standard	3 and 3 (appendix)
Analysis	**14**	Methods for estimating or comparing measures of diagnostic accuracy	3; 4
	**15**	How indeterminate index test or reference standard results were handled	3 and 2;3;4 (appendix)
	**16**	How missing data on the index test and reference standard were handled	4
	**17**	Any analyses of variability in diagnostic accuracy, distinguishing pre-specified from exploratory	not applicable
	**18**	Intended sample size and how it was determined	3
**RESULTS**			
Participants	**19**	Flow of participants, using a diagram	4; Figure 1
	**20**	Baseline demographic and clinical characteristics of participants	4; Figure 2
	**21a**	Distribution of severity of disease in those with the target condition	not applicable
	**21b**	Distribution of alternative diagnoses in those without the target condition	not applicable
	**22**	Time interval and any clinical interventions between index test and reference standard	2
Test results	**23**	Cross tabulation of the index test results (or their distribution) by the results of the reference standard	6; 7; 8;9
	**24**	Estimates of diagnostic accuracy and their precision (such as 95% confidence intervals)	6; 7; 8;9
	**25**	Any adverse events from performing the index test or the reference standard	not applicable
**DISCUSSION**			
	**26**	Study limitations, including sources of potential bias, statistical uncertainty, and generalisability	11
	**27**	Implications for practice, including the intended use and clinical role of the index test	10;11
**OTHER INFORMATION**			
	**28**	Registration number and name of registry	11
	**29**	Where the full study protocol can be accessed	11
	**30**	Sources of funding and other support; role of funders	11

References

1. Corman VM, Landt, O., Kaiser, M.; et al. Detection of 2019 novel coronavirus (2019-nCoV) by real-time RT-PCR. *Eurosurveillance* **2020**, *25*, 2000045.
2. Rutjes AWS, Reitsma JB, Coomarasamy A, Khan KS, Bossuyt PMM. Evaluation of diagnostic tests when there is no gold standard. A review of methods. *Health Technol. Assess. (Rockv)*. **2007**, *11*.
3. Reitsma JB, Rutjes AWS, Khan KS, Coomarasamy, A., Bossuyt PM. A review of solutions for diagnostic accuracy studies with an imperfect or missing reference standard. *J. Clin. Epidemiol*. **2009**, *62*, 797-806.
4. Dendukuri N, Schiller I, De Groot, J., et al. Concerns about composite reference standards in diagnostic research. *BMJ* **2018**, *360*, j5779.
5. Goetghebeur E, Liinev J, Boelaert, M., van P. Diagnostic test analyses in search of their gold standard: Latent class analyses with random effects. *Stat. Methods Med. Res*. **2003**, *9*, 231-48.
6. Pepe MS and Alonzo TA. Reply to Letter to Editor regarding Alonzo TA and Pepe MS, Assessing the accuracy of a new diagnostic test when a gold standard does not exist. *Statistics in Medicine*. **2001**, *20*, 656-660.

© 2020 by the authors. Submitted for possible open access publication under the terms and conditions of the Creative Commons Attribution (CC BY) license (http://creativecommons.org/licenses/by/4.0/).
